# Selenized Plant Oil Is an Efficient Source of Selenium for Selenoprotein Biosynthesis in Human Cell Lines

**DOI:** 10.3390/nu11071524

**Published:** 2019-07-04

**Authors:** Jordan Sonet, Maurine Mosca, Katarzyna Bierla, Karolina Modzelewska, Anna Flis-Borsuk, Piotr Suchocki, Iza Ksiazek, Elzbieta Anuszewska, Anne-Laure Bulteau, Joanna Szpunar, Ryszard Lobinski, Laurent Chavatte

**Affiliations:** 1 CNRS/UPPA, Institute of Analytical and Physical Chemistry for the Environment and Materials (IPREM), UMR5254, Hélioparc, F-64053 Pau, France; 2 Department of Bioanalysis and Analysis of Drugs, Medical University of Warsaw, Żwirki i Wigury 81, 02-091 Warszawa, Poland; 3 Department of Biochemistry and Biopharmaceuticals, National Medicines Institute, Chelmska 30/34, PL-00-725 Warszawa, Poland; 4 Institut de Génomique Fonctionnelle de Lyon, IGFL, CNRS/ENS UMR5242, 69007 Lyon, France; 5 Centre International de Recherche en Infectiologie, CIRI, 69007 Lyon, France; 6 Institut national de la santé et de la recherche médicale (INSERM) Unité U1111, 69007 Lyon, France; 7 Centre national de la recherche scientifique (CNRS), Ecole Normale Supérieure (ENS) de Lyon, Université Claude Bernard Lyon 1 (UCBL1), Unité Mixte de Recherche 5308 (UMR5308), 69007 Lyon, France

**Keywords:** selenium, selenoprotein, selenized lipids, Selol, Gpx1, Gpx4, Txnrd1, Txnrd2, ICP-MS

## Abstract

Selenium is an essential trace element which is incorporated in the form of a rare amino acid, the selenocysteine, into an important group of proteins, the selenoproteins. Among the twenty-five selenoprotein genes identified to date, several have important cellular functions in antioxidant defense, cell signaling and redox homeostasis. Many selenoproteins are regulated by the availability of selenium which mostly occurs in the form of water-soluble molecules, either organic (selenomethionine, selenocysteine, and selenoproteins) or inorganic (selenate or selenite). Recently, a mixture of selenitriglycerides, obtained by the reaction of selenite with sunflower oil at high temperature, referred to as Selol, was proposed as a novel non-toxic, highly bioavailable and active antioxidant and antineoplastic agent. Free selenite is not present in the final product since the two phases (water soluble and oil) are separated and the residual water-soluble selenite discarded. Here we compare the assimilation of selenium as Selol, selenite and selenate by various cancerous (LNCaP) or immortalized (HEK293 and PNT1A) cell lines. An approach combining analytical chemistry, molecular biology and biochemistry demonstrated that selenium from Selol was efficiently incorporated in selenoproteins in human cell lines, and thus produced the first ever evidence of the bioavailability of selenium from selenized lipids.

## 1. Introduction

**Figure 1:**
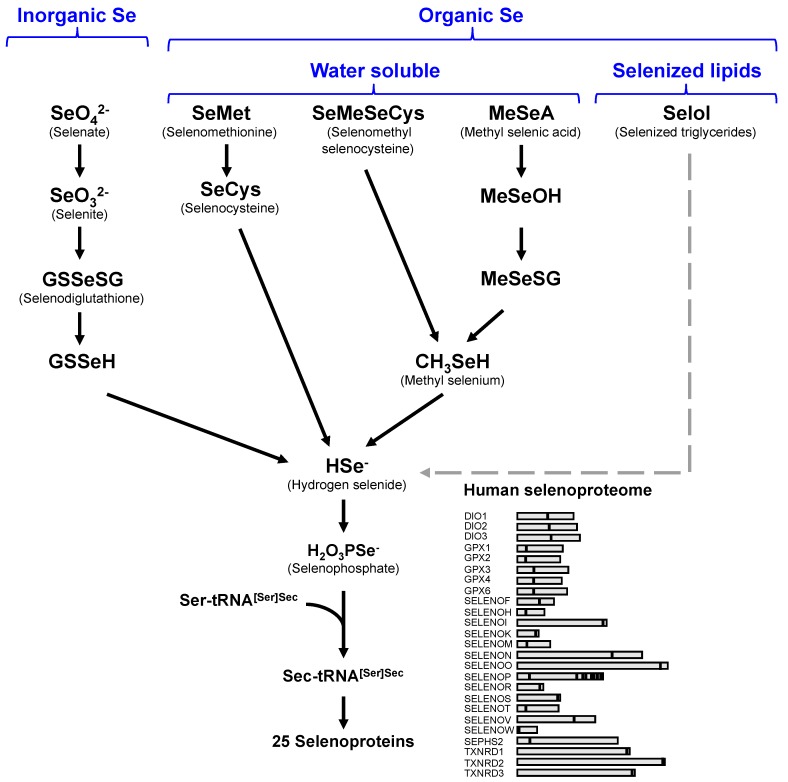
Representation of the various selenium metabolic pathways leading to selenoprotein synthesis. So far, every metabolic pathway towards selenoproteins converges to selenide. The dashed line suggest a putative metabolic pathway for selenium assimilation from selenized lipids.

Selenocysteine is a rare amino acid which is vital for the function of selenoproteins, a unique group of genetically encoded proteins in redox reactions. Twenty-five selenoprotein genes have been identified in the human genome [1]. Selenoprotein synthesis depends upon Se intake, Se transport and Se conversion to selenocysteine (Sec) and its co-translational insertion into selenoproteins [2,3]. Several chemical forms, including selenomethionine, selenocysteine, selenite, and selenate, account for almost all the selenium in diets. All these forms are absorbed without regulation, and all have high bioavailability [4,5]. Among them, selenomethionine accounts for 90% of the total selenium in plants and is randomly incorporated into proteins at methionine positions. Free selenomethionine is easily available through the digestion of dietary proteins. Selenocysteine can also be a selenium source from animal proteins. However, the highly reactive free selenocysteine is maintained at very low concentrations in tissues. Although inorganic selenium compounds (selenite, and selenate) are less abundant than selenomethionine, they are an efficient source of selenium for selenoprotein biosynthesis. Selenate is reduced to selenite prior to its utilization as a selenium source. Then, selenite is reduced to selenide (HSe^-^), which is the central precursor for selenoprotein biosynthesis (see Figure 1). All selenocompounds need to be metabolized in selenide in order to be incorporated in genetically encoded selenoproteins. Additionally, among the selenometabolites, which are mostly excreted in urine, only a selenosugar (1β-methylseleno-N-acetyl-D-galactosamine) is, to a certain extent, bioavailable for selenoprotein biosynthesis when injected intravenously in animals [6].

Like for other essential trace elements, the selenium beneficial effect in cells follows a bell-shaped curve, a deficiency or an excess leading to toxicity and eventually lethality [7]. In cell lines, the window of benefit varies widely from one form of selenium to another and from one cell to another [5,8,9]. For example, in prostate cells exposed to selenite, the stimulation of selenoprotein expression is observed in the nanomolar range and toxicity starts at micromolar concentrations [10]. In contrast to that, selenate and selenomethionine are less toxic than selenite and the bell-shaped curve is shifted to higher concentrations. In liver cell lines, a comparative study with seven selenocompounds focusing on relative bioactivity and toxicity revealed highly variable behavior with regards to the chemical selenium form added to the culture media [4]. Again, at low doses, selenite showed a higher potential to activate selenoprotein synthesis than selenate; a similar shift in toxicity concentration was observed. 

In genetically encoded selenoproteins, selenocysteine insertion occurs at UGA codons, which are normally used as a termination signal, and therefore follows a non-conventional mechanism [2,3,11,12,13,14]. To circumvent the reading of UGA as a stop signal, selenoprotein mRNAs have a Sec insertion sequence (SECIS) located in the 3’ untranslated region (UTR). This SECIS serves as a platform to recruit the recoding factors for selenocysteine insertion, to deliver the Sec-tRNA^[Ser]Sec^ to the ribosomal A site. Interestingly, the SECIS element is necessary and sufficient to recode every in-frame UGA in a heterologous system. This feature has been particularly useful to characterize the functional determinants of the SECIS element in reporter constructs. Indeed a luciferase-based reporter construct that is stably expressed in HEK293 cells, in which the SECIS element of glutathione peroxidase-4 (Gpx4) is cloned downstream of the firefly luciferase containing an in frame UGA, was developed. When the UGA is read as a stop codon, the truncated luciferase is inactive, but when the UGA is read as a selenocysteine, the luciferase is fully active. The recoding efficiency is directly correlated to the luciferase activity measured in cellular extracts [15,16]. This reporter construct has been powerful to evaluate the variation of selenocysteine insertion efficiency in response to various stimuli which include selenium level variation, oxidative stress, and replicative senescence. 

**Figure 2:**
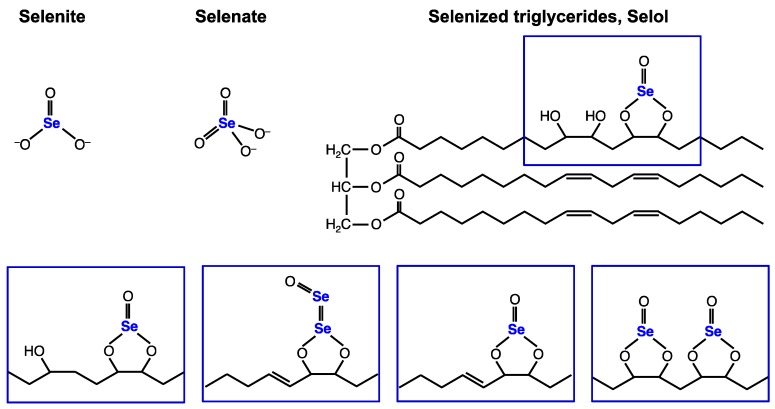
Chemical structures of selenite, selenate and Selol. In Selol, a complex mixture of at least 11 selenitriglyceride compounds have been characterized by high performance liquid chromatography-electrospray ionization tandem mass spectrometry (HLPC-ESI MS^n^) [19].

The search for bioavailable selenocompounds with lower toxicity than selenite drove research to design a selenized triglyceride, which is produced by the reaction of selenite with sunflower oil. This compound referred to as Selol is currently investigated for its potential anticancer properties and antineoplastic effects [17,18,19,20,21]. The reaction of sunflower oil with selenite generates a complex mixture of selenitriglycerides, which has been characterized recently by high performance liquid chromatography-inductively coupled plasma mass spectrometry (HPLC-ICP MS) and high performance liquid chromatography-electrospray ionization tandem mass spectrometry (HPLC ESI MS/MS). A total of 11 selenium-containing triglycerol derivatives have been identified where selenium is in the [Se(IV)] oxidation state [19], see Figure 2.

However, the bioavailability of Selol for selenoprotein biosynthesis has not been investigated yet. The goal of this work was to investigate the assimilation of selenium from Selol and to compare it with that of inorganic species (selenite and selenate) in various cell lines, which are relevant for selenium physiological function. We are reporting here for the first time that a selenized lipid could be an efficient source of selenium for selenoprotein biosynthesis in HEK293 and LNCaP cell lines, which originate from embryonic kidney tissue and prostate adenocarcinoma, respectively. 

## 2. Materials and Methods

### 2.1. Materials

The HEK293, LNCaP and PNT1A cells line used in this study were purchased from Life Technologies (Carlsbad, CA, USA, Cat.# R75007 and R71407), ATCC (Manassas, VA, USA, Cat.# CRL-1740) and SIGMA (Saint-Louis, MO, USA, Cat.# 95012614), respectively. The HEK293 cell line expressing Luc UGA/Gpx4 in a stable way was generated and validated in References [15,16,22]. Fetal calf serum (FCS), cell culture media and supplements were purchased from Life Technologies. Transferrin, insulin, 3,5,3′-triiodothyronine, hydrocortisone, sodium selenite and sodium selenate were purchased from Sigma-Aldrich (Saint-Louis, MO, USA). Antibodies were purchased from Abcam (Cambridge, UK) (glutathione peroxidase-1 (Gpx1), #ab108429; glutathione peroxidase-4 Gpx4, #ab125066; thioredoxin reductase-1 (TxnRD1), #ab124954) and Sigma-Aldrich (thioredoxin reductase-2 (Txnrd2), #HPA003323; Actin, #A1978). NuPAGE 4-12% bis–Tris polyacrylamide gels and MOPS SDS running buffer were purchased from Life Technologies. Antiprotease inhibitor cocktail was purchased from Thermo Fisher. Selol was synthesized at the Department of Bioanalysis and Drug Analysis at the Medical University of Warsaw, as described in Reference [23]. A micellar solution of Selol was used (based on lecithin, water and Selol) with a declared selenium concentration of 5% (w/v). 

### 2.2. Cell Culture and Incubation with Different Forms of Selenium

HEK293 and LNCaP were grown and maintained in D-MEM, while RPMI medium was used for PNT1A. Media were supplemented with 10% FCS. Cells were cultivated at 37 °C and in a humidified atmosphere containing 5% CO_2_. Since the selenium is endogenously provided by the FCS, the same lot number was kept throughout the experiments. Selenium concentration (194 nM) was determined by ICP MS in an FCS lot number (41G7530K) as reported in Reference [24]. Two different culture media, referred to as control (Ctrl) and depleted (Dpl) were used according to Reference [16]. Selenium concentration in the Ctrl medium was 19.4 nM. In the Dpl medium, we expected 3.9 nM selenium because 2% FCS was used instead of 10%. To cope with the decrease of growth factors in Dpl medium, 5 mg/L transferrin, 10 mg/L insulin, 100 pM 3,5,3’-triiodothyronine, and 50 nM hydrocortisone were added, as described and validated previously for selenoprotein expression studies. 

The dose–response experiment with selenite, selenate and SELOL was performed with HEK293 cells stably expressing the Luc UGA/Gpx4 construct. Different media containing the respective concentrations of selenocompounds were prepared in Dpl media and used to cultivate the HEK293 cells for three days in a 10 cm (diameter) culture dish. After the treatment, cellular extracts were harvested with a volume of 300 µL of passive lysis buffer containing 25 mM Tris phosphate pH 7.8, 2 mM DTT, 2 mM EDTA, 1% Triton X100 and 10% glycerol. Then, protein concentrations were measured using the DC kit protein assay kit (Biorad, Hercules, CA, USA) in microplate assays using the microplate reader FLUOstar OPTIMA (BMG Labtech, Champigny-sur-Marne, France). Morphological evaluation was performed by phase-contrast microscopy using an Evos microscope (Ozyme, Saint-Cyr-l’École, France) with a 10X objective.

The comparative treatment of HEK293, LNCaP and PNT1A was performed in Ctrl medium supplemented or not with 100 nM or 100 µM of selenocompounds (selenite, selenate or Selol). Cells were grown for three days in 10 cm diameter culture dish.

### 2.3. Measure of Se Levels by ICP-MS

Flow injection–inductively coupled plasma mass spectrometry (FI-ICP-MS) methodology was used to measure total levels of selenium as reported in Reference [24]. Cellular extracts (200 μL) were mixed with 3 μL of nitric acid (70%) and left at room temperature overnight. Plasma conditions and detection parameters were optimized with 1 ppb Y, Li, Tl, and Ce in 2% of nitric acid. Signals of ^77^Se, ^78^Se, and ^80^Se were acquired. A calibration curve was prepared from 5 ppm Se solution (calibration points: 1, 2.5, 5, 10, 25, 50, 100 ppb Se in 1 mL HNO_3_, 1%). An Agilent (Santa Clara, CA, USA) 7500 ICP MS instrument fitted with a reaction/collision cell (H_2_) was used. Every sample was analyzed by three consecutive injections of 50 µL.

### 2.4. Evaluation of Selenocysteine Insertion Efficiency

**Figure 3:**
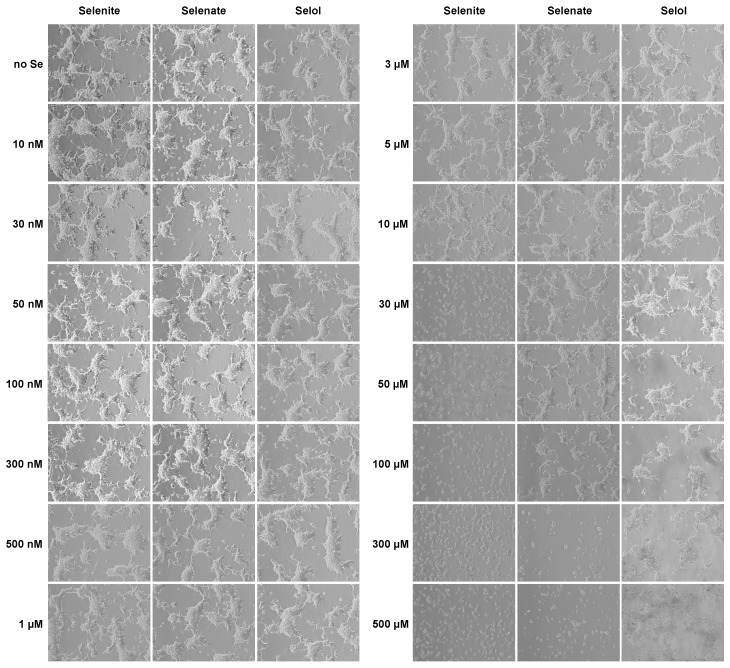
HEK293 morphology in response to various concentrations of three selenocompounds, namely selenite, selenate and Selol. Cells were grown for three days as described in materials and methods. Toxic effect of selenium is visible by cell detachment from the culture dish.

To analyze Sec insertion efficiency in HEK293, we used luciferase-based reporter constructs which were validated for UGA/Sec recoding in transfected cells [22,25]. Briefly, the minimal SECIS element from Gpx4 was cloned downstream of a luciferase coding sequence, which was modified to contain an in frame UGA codon at position 258 (Luc UGA/SECIS), as shown in Figure 3. HEK293 cells stably expressing Luc UGA/Gpx4 SECIS were previously generated and validated [15,16]. After growth in the presence of various concentrations of sodium selenite, cells were harvested and the cellular extracts were assayed for luciferase activity by chemiluminescence (luciferase assay systems, Promega, Madison, WI, USA), in triplicate using a microplate reader FLUOstar OPTIMA (BMG Labtech). We expressed the Sec insertion efficiency relative to the luciferase activity measured in Dpl conditions arbitrarily, setting it at 1.

### 2.5. Protein Gels and Western Immunoblotting

Equal protein amounts (20 µg) were separated in Bis-Tris NuPAGE Novex Midi Gels and transferred onto nitrocellulose membranes using iBlot® dry blotting system (Life Technologies). Membranes were probed with primary antibodies (as indicated) and HRP-conjugated anti-rabbit or anti-mouse secondary antibodies (Sigma, #A9044 and #A6154, respectively). The chemiluminescence signal was detected using the ECL select Western blotting detection kit (GE Healthcare, Chicago, IL, USA) and the PXi 4 CCD camera (Ozyme). Image acquisition and data quantifications were performed with the Syngene softwares, GeneSys and Genetools, respectively.

## 3. Results

### 3.1. Comparison of the Selenium Uptake by HEK293 from Selol, Selenite and Selenate

The chemical structures of selenite, selenate and Selol are represented in Figure 2. Selenite and selenite differ in terms of their capacity to stimulate selenoprotein expression but also in terms of their active and toxic concentration ranges. In Selol, selenium thought to be at the oxidation state IV as in selenite, seems to be much less toxic than selenite in fibroblasts and prostate cells [23]. We investigated first the potential of these three selenocompounds to be taken up by HEK293 cells in a wide range of concentrations, by measuring the total selenium by ICP-MS. Dpl culture media containing 15 different concentrations of selenium (from 10 nM to 500 µM) in the form of selenite, selenate and Selol were prepared and used to grow HEK293 for three days. First, HEK293 cells were observed with a phase-contrast optical microscope and compared to the Dpl conditions without selenium (Figure 3). The toxicity of selenium was easily noticed using a microscope at the morphological level by the loss of cell attachment to the culture dish. As shown in Figure 3, this lack of attachment was observed at 30 µM and higher concentrations of selenite, but only at 300 µM and higher concentrations of selenate in the culture media.

**Figure 4:**
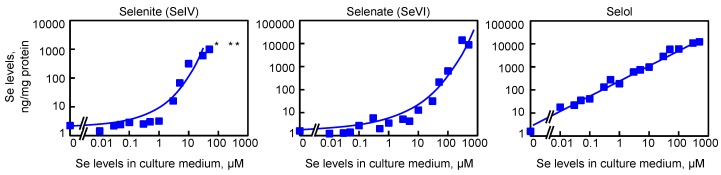
Measurement of selenium levels of HEK293 cells grown with increasing concentration of selenite, selenate or Selol in the culture media (from 10 nM up to 500 µM). Selenium levels are expressed relative to protein concentration. In several extracts, due to selenite toxicity, the protein recovery was too low to allow precise selenium detection and normalization, as indicated by an asterisk.

Then, the cells (adherent and detached) grown in various selenium conditions were harvested and the cellular extracts were analyzed for the total selenium concentration by FI-ICP-MS. Selenium levels were normalized for the protein concentration, and plotted as a function of the selenium concentration in the culture medium, as illustrated in Figure 4. With the selenite concentrations in the culture medium of 100 µM and higher, the protein recovery was too low to allow the precise selenium detection (asterisk in Figure 4). Interestingly, we noticed that levels of selenium were increased at a maximum by three orders of magnitude to reach approximately 1 µg Se per mg of proteins. However, this increase was not a linear function of the selenium concentration in the growth medium. As shown in Figure 4, with selenate, a similar trend was observed, although shifted to higher concentrations. The loss of cell attachment started at a concentration of 300 µM in the culture media. Concerning Selol, a different behavior was observed with a linear relationship (at a log scale) between the assimilated selenium and the selenium added to the culture medium. With the maximal concentration of Selol tested here (500 µM), no sign of cell detachment was observed (Figure 3) and the Se concentration reached 12 µg per mg of proteins (Figure 4).

### 3.2. Selol is Able to Stimulate UGA Recoding as Selenocysteine in HEK293 Cells

**Figure 5:**
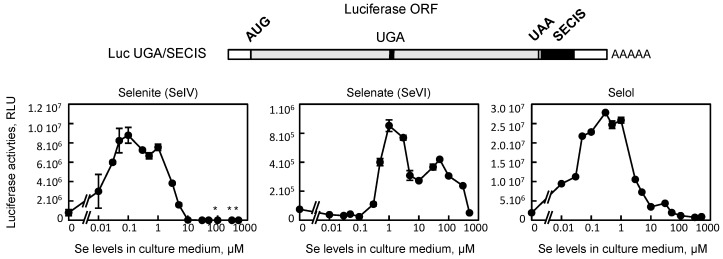
Evaluation of selenocysteine insertion efficiency in response to selenium supplementation in HEK293 cells. The luciferase activities from the stably expressing cell lines are measured from cells grown in increasing concentration of selenite, selenate or Selol in the culture media (from 10 nM up to 500 µM). Luciferase activities are expressed relative to protein concentration (relative luciferase unit, RLU). In several extracts, due to selenium toxicity, the protein recovery is too low to allow precise selenium detection and normalization, as indicated by an asterisk. A schematic of the luciferase construct is shown on top of histograms. AUG, start codon; UGA, selenocysteine codon; UAA, stop codon; SECIS, selenocysteine insertion sequence.

The HEK293 cell line used in this study was genetically modified by a stably expressing luciferase-based reporter construct. The latter was validated previously to evaluate selenoprotein expression by measuring the efficiency of UGA recoding as selenocysteine [15,25,26]. Briefly, the minimal SECIS elements from Gpx4 3’UTR was cloned downstream of a luciferase coding sequence, which had been modified to contain an in frame UGA codon at position 258 (Luc UGA/SECIS), as shown in Figure 5. The luciferase activity was particularly sensitive to the Se supplementation of the culture, as shown previously with selenite [16]. The cellular extracts from cells grown in various concentrations of selenite, selenate and Selol were also evaluated for their luciferase activities. The enzymatic activities were normalized for the protein concentration and expressed relatively to the Dpl conditions, set as 1. As it has been mentioned before, an important stimulation of selenocysteine insertion was observed with low concentrations of selenite to reach a plateau at 100 nM up to 1 µM (see Figure 4). With higher doses of selenite in the culture medium, a rapid decrease of UGA recoding efficiency occurred to be almost undetectable at 10 µM. Interestingly, this decrease in selenocysteine insertion between 1 and 10 µM of selenite correlated with the massive increase in selenium levels (cf. graphs left panels in Figure 4 and Figure 5). These data suggest that the level of selenium is tightly linked with the optimal selenocysteine insertion, and that overwhelming selenium levels seem to shut down the selenoprotein expression at a high dose. Alternatively, when selenate was used to supplement cell culture medium instead of selenite, a similar stimulation of UGA recoding efficiency was observed but shifted to higher concentrations. The plateau was reached at 1 µM of selenate and started decreasing at 100 µM. Again, when selenium levels and selenocysteine insertion efficiency were compared at high selenate concentrations (cf. Figure 4 and Figure 5), we observed a similar inverse correlation as with selenite. These data confirm further that an excess of intracellular inorganic selenium could be detrimental to the cell integrity. Then, we investigated whether Selol was able to modulate the selenocysteine insertion efficiency. As shown in Figure 5, strong stimulation of UGA recoding activities occurred when Selol was added to the culture medium even at the lowest concentration tested (10 nM). A plateau of luciferase activity was reached at 100 nM and lasted up to 1 µM, similarly to selenite. Then, at higher concentrations, the recoding efficiency decreased slowly to reach the initial activity. These results demonstrate clearly for the first time the bioavailability of selenium bound to triglyceride for the selenocysteine insertion into proteins.

### 3.3. Selol Upregulates Selenoprotein Expression Also in LNCaP but not in PNT1A

**Figure 6:**
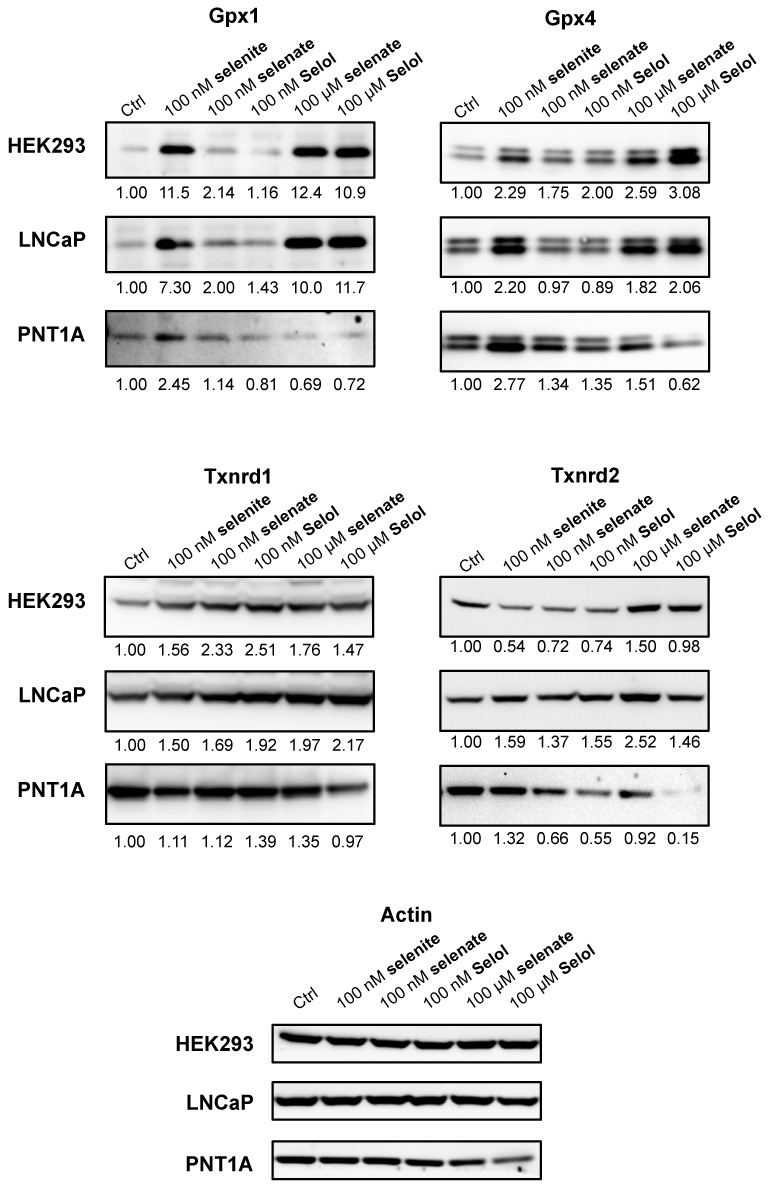
Analysis of selenoprotein regulation by selenite, selenate or Selol supplementation in different cell lines. HEK293, LNCaP and PNT1A cells were grown with Ctrl medium supplemented with either 100 nM or 100 µM of different selenium sources. Cellular extracts were analyzed for selenoprotein expression by western blots using specific antibodies. Protein levels were normalized over Actin levels and expressed relative to the Ctrl condition (no added selenium), set as 1.

**Figure 7:**
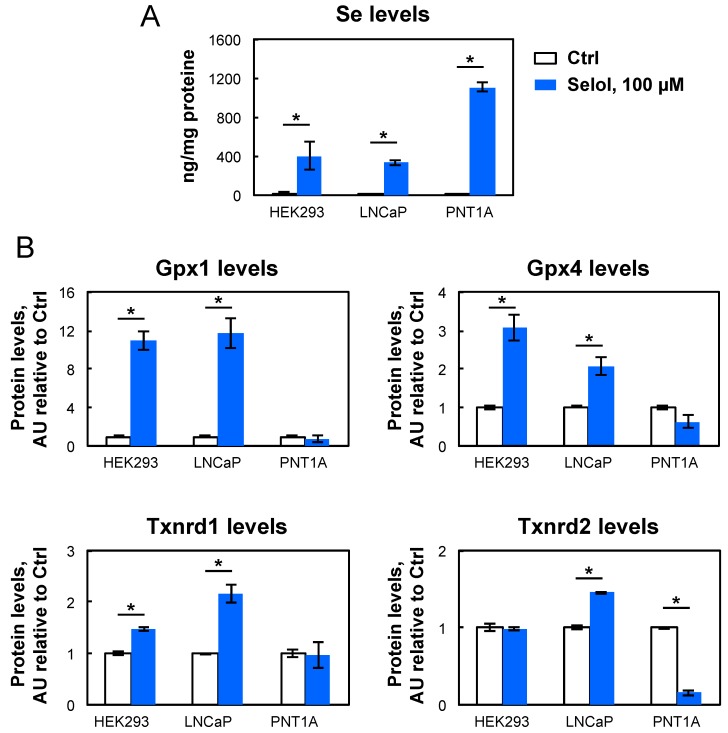
Comparison of Selol activities in three different human cell lines. (**A**) Selenium content in HEK293, LNCaP, PNT1A treated or not with 100 µM Selol. (**B**) Quantification of selenoprotein expression levels detected in Figure 6, normalized over the actin signal, in response to Selol supplementation. The significantly different (student test, *p* < 0.01) variations are indicated with an asterisk above the respective bars.

Then, we investigated whether Selol was able to stimulate selenoprotein expression in prostate cell lines since this source of selenium was considered as a potential antineoplastic drug in prostate cancer. Here, we used LNCaP and PNT1A prostate cell lines, which are cancerous or immortalized, respectively. The LNCaP cells were previously found to be more sensitive than PNT1A to selenium-induced toxicity, both with selenite and Selol [23]. Here, we worked at similar selenium concentrations (i.e., 100 nM and 100 µM) with these cell lines as with HEK293 cells. As illustrated in Figure 6 and Figure 7, the response of LNCaP to selenium supplementation was very similar to that of HEK293 cell lines; a strong stimulation of Gpx1 and Gpx4 was observed with 100 nM of selenite, and with 100 µM of selenate or Selol. Our data further support the bioavailability of Selol for selenoprotein biosynthesis. For the housekeeping selenoproteins, Selol (used at 100 nM or 100 µM) was also able to upregulate significantly Txnrd1 and Txnrd2 in LNCaP, similarly to selenite and selenate. On the other hand, PNT1A appeared poorly sensitive to selenium level variations. Interestingly, only the addition of 100 nM selenite to the culture medium had a moderate effect on Gpx1 and Gpx4, selenate and Selol being ineffective. 

To confirm further that Selol has a cell line specific effect on selenoprotein expression, we verified that the differences observed between these cell lines did not result from a difference in selenium intake. For that, selenium levels were measured in the presence of 100 µM Selol (Figure 7, top histogram). We found that the different cell lines tested in our study were equally competent to take up selenium from Selol. In addition, our data suggest that the lack of selenoprotein upregulation by Selol in PNT1A may result from a difference in the capacity to efficiently metabolize Selol in an active precursor of Sec-tRNA^[Ser]Sec^, than in other cell lines such as HEK293 or LNCaP.

## 4. Discussion

In the environment, Se is present in many chemical forms and at variable concentrations. As selenium shares many chemical properties with sulfur, virtually any sulfur-containing molecule can have its analog with selenium instead [5,27]. This has been particularly observed in organisms such as higher plants and yeast that do not require selenium but are grown in a selenium-rich environment. The common strategy to detoxify this element is to incorporate selenium into organic molecules. However, to our knowledge, no selenized-lipid molecule has been observed so far in the wide diversity of the natural organic selenium species [27]. Among the selenodrugs that have been designed and synthesized, mostly for antioxidant and anticancer properties [28], Selol is a mixture of selenized triglycerides obtained by the reaction of Se_+IV_ with sunflower oil, in which at least 11 different selenium-containing triglycerol derivatives were characterized by mass spectrometry [19]. Here, we demonstrated that the selenium present in these compounds was efficiently bioavailable for human cell lines. It is widely accepted that the genetically encoded selenoproteins represent the biologically active form of selenium in mammalian cells. Among the twenty-five selenoproteins, many are involved in antioxidant defense, redox homeostasis and redox signaling. The selenoproteome is composed of five glutathione peroxidases (Gpx1-Gpx4, Gpx6), three thioredoxin reductases (Txnrd1-Txnrd3), one methionine sulfoxide reductase (MsrB1) and seven selenoproteins located in the endoplasmic reticulum (Dio2, SelenoF, SelenoK, SelenoM, SelenoN, SelenoS and SelenoT). However, about half of the selenoproteome remains without a precise function [3]. In the selenoproteome, Gpx1, Gpx4, Txnrd1 and Txnrd2 are ubiquitously expressed and react differently to the variation of selenium level. That is why we compared the potential of Selol to stimulate these selenoproteins with selenite and selenate, which are currently used for supplementation studies. Our main finding was that human cells use selenium from a mixture of selenized lipids for the synthesis of selenoproteins. One significant difference of Selol with other selenium compounds was the cell line specific activities. Indeed, Selol appeared to indifferently enter the three cell lines studied here but its use for selenoprotein synthesis was only efficient in HEK293 and LNCaP, but not in PNT1A which are non-cancerous prostate cell lines. This selectivity can come from a difference in lipid metabolism between the different cell lines studied in the present work. Indeed, as illustrated in Figure 2 and discussed in detail in Reference [5], every chemical selenium species that enters the cell has to be transformed into selenide to be further incorporated into selenoproteins. Three metabolic routes have been identified so far to generate hydrogen selenide, namely from selenite, selenocysteine, and methylselenol. How selenite is released from the lipids and whether all selenized triglycerides are equally competent for this release awaits further investigation. One possibility is that selenium is made bioavailable by lipolysis of the triglycerides followed by beta-oxidation of fatty acids.

Investigations about Selol mostly concern its anticancer activities [17,18,19,20,21,23,29], very little work has been done on antioxidant activities and potential use for not cancerous cells. The present study demonstrated the high level of selenium assimilation from this mixture of selenized-lipids and confirmed the lower toxicity in comparison to other selenocompounds. The cellular selectivity for insertion into selenoproteins has to be extended to other cellular models, either cancerous or not, to get a better picture of whether this new compound can be used as a dietary supplement.

## 5. Conclusions

This study is, to our knowledge, the first report for assimilation of Se from lipids into selenoproteins. Whether selenized lipids are present in the human food diet is an open issue due to the inherent experimental detection and characterization difficulties. Clearly, for the cell lines tested so far, Selol is much less toxic than selenite and selenate. Now being tested for antineoplastic activity, it may be considered as a potential nutritional source of selenium for deficient populations. Similarly to selenite and selenate, Selol is able to upregulate selenoprotein synthesis by following a precise prioritization of selenium use, also referred to as selenoprotein hierarchy [2,3]. Selol has a cell line specific capacity to upregulate selenocysteine insertion. This selectivity can be due to a cell line specific ability to metabolize Selol to make selenium bioavailable, most probably by lipolysis and beta-oxidation of fatty acid pathways. Despite similar uptake, the metabolism of Selol in selenide is different in PNT1A from HEK293 or LNCaP cells.
